# Supplementary material for: Modeling the ACVR1R206H mutation in human skeletal muscle stem cells
Source: eLife. 2021 Nov 10;10:e66107. doi: 10.7554/eLife.66107 (PMC8691832; doi:10.7554/eLife.66107)
Supplement: Figure 1—source data 1. [file elife-66107-fig1-data1.docx]

| **Figures** | **Muscle** | **Age** | **Sex** | **Subjects** |
| --- | --- | --- | --- | --- |
| Figure 1A | Psoas | 52 | F | FOP |
| Figure 1A | Intercostal | 46 | F | FOP |
| Figure 1B (top) | Vastus Lateralis | 59 | M | Control |
| Figure 1B (middle) | Biceps Brachii | 21 | F | FOP |
| Figure 1B (bottom) | Biceps Brachii | 32 | F | FOP |
| Figure 1C-H and S1 | Biceps Brachii | 44 | F | Control |
| Figure 1C-H and S1 | Biceps Brachii | 32 | F | FOP |
| Figure 1C-H and S1 | Diaphragm | 32 | F | FOP |

**Figure 1-Source Data 1. Muscle specimen information.**
