# Supplementary material for: Modeling the ACVR1R206H mutation in human skeletal muscle stem cells
Source: eLife. 2021 Nov 10;10:e66107. doi: 10.7554/eLife.66107 (PMC8691832; doi:10.7554/eLife.66107)
Supplement: Figure 1—source data 2. [file elife-66107-fig1-data2.docx]

| **Week 5** |  |  |  |
| --- | --- | --- | --- |
| Satellite Cells isolated from | # of cells transplanted/mouse | Human Dystrophin fibers | Human PAX7+ cells |
| Ctrl Biceps | 6000 | 38 | 4 |
| Ctrl Biceps | 6000 | 30 | 7 |
| Ctrl Biceps | 6000 | 20 | 1 |
| FOP Diaphragm | 5000 | 13 | 1 |
| FOP Diaphragm | 5000 | 8 | 1 |
| FOP Diaphragm | 5000 | 6 | 0 |
| FOP Biceps | 2800 | 5 | 3 |
| FOP Biceps | 2800 | 5 | 2 |
| FOP Biceps | 2800 | 0 | 2 |
| **Week10** |  |  |  |
| Ctrl Biceps | 6000 | 133 | 24 |
| Ctrl Biceps | 6000 | 112 | 13 |
| Ctrl Biceps | 6000 | 82 | 19 |
| FOP Diaphragm | 5000 | 98 | 23 |
| FOP Diaphragm | 5000 | 120 | 22 |
| FOP Diaphragm | 5000 | 57 | 10 |
| FOP Diaphragm | 5000 | 42 | 12 |
| FOP Biceps | 2800 | 0 | 0 |
| FOP Biceps | 2800 | 58 | 15 |
| FOP Biceps | 2800 | 12 | 5 |

**Figure 1-Source Data 2. Hu-MuSCs transplantation details.**
