# Supplementary material for: Modeling the ACVR1R206H mutation in human skeletal muscle stem cells
Source: eLife. 2021 Nov 10;10:e66107. doi: 10.7554/eLife.66107 (PMC8691832; doi:10.7554/eLife.66107)
Supplement: Figure 3—source data 1. [file elife-66107-fig3-data1.docx]

| **Cell line** | **# of cells transplanted** | **Human Dystrophin fibers** | **Human PAX7+ cells** |
| --- | --- | --- | --- |
| WTC11 | 8000 | 6 | 0 |
| WTC11 | 8261 | 0 | 0 |
| WTC11 | 12341 | 0 | 0 |
| 1323-2 | 8011 | 0 | 0 |
| 1323-2 | 7873 | 0 | 0 |
| BJ2 | 15000 | 2 | 0 |
| BJ2 | 887 | 0 | 0 |
| BJ2 | 9767 | 1 | 0 |
| F1-1 | 5508 | 0 | 0 |
| F1-1 | 6084 | 0 | 0 |
| F1-1 | 15221 | 0 | 0 |
| F2-3 | 17638 | 15 | 2 |
| F3-2 | 30000 | 51 | 0 |
| F3-2 | 1300 | 0 | 0 |
| F3-2 | 40000 | 10 | 0 |

**Figure 3-Source Data 1: hiPSC-derived HNK1^-^CD45^-^CD31^-^ CXCR4^+^CD29^+^CD56^dim^ cell transplants.**
