# Supplementary material for: Modeling the ACVR1R206H mutation in human skeletal muscle stem cells
Source: eLife. 2021 Nov 10;10:e66107. doi: 10.7554/eLife.66107 (PMC8691832; doi:10.7554/eLife.66107)
Supplement: Figure 4—source data 1. [file elife-66107-fig4-data1.docx]

|  | **Control (1323-2)** | **FOP (F3-2)** |
| --- | --- | --- |
| Estimated Number of Cells | 2,054 | 5,306 |
| Mean Reads per Cell | 76,178 | 32,870 |
| Median Genes per Cell | 2,689 | 2,501 |
| Number of Reads | 156,470,954 | 174,413,305 |
| Valid Barcodes | 97.8% | 98.0% |
| Reads Mapped Confidently to Transcriptome | 63.1% | 66.3% |
| Reads Mapped Confidently to Exonic Regions | 67.0% | 70.3% |
| Reads Mapped Confidently to Intronic Regions | 17.4% | 15.3% |
| Reads Mapped Confidently to Intergenic Regions | 3.6% | 3.4% |
| Reads Mapped to Antisense to Gene | 4.7% | 4.9% |
| Sequencing Saturation | 79.0% | 54.8% |
| Fraction Reads in Cells | 21,100 | 22,238 |

**Figure 4-Source Data 1:** **Quality control information for each sample.**
